# Sex-Specific Associations Between Smokeless Tobacco Use and *Staphylococcus aureus* Carriage in Norwegian Adolescents

**DOI:** 10.1177/1179173X261461691

**Published:** 2026-06-24

**Authors:** Dina B. Stensen, Anna Karlsen, Gunnar Skov Simonsen, Anne-Sofie Furberg

**Affiliations:** 1 Department of Microbiology and Infection Control, 60519 University Hospital of North Norway, Tromsø, Norway; 2 Department of Ophthalmology, 60519 University Hospital of North Norway, Tromsø, Norway; 3 Department of Medical Biology, Faculty of Health Sciences, 8016 UiT The Arctic University of Norway, Tromsø, Norway; 4 Faculty of Health and Social Sciences, 5562 Molde University College, Molde, Norway

**Keywords:** *Staphylococcus aureus*, colonization, carriage, tobacco, smokeless

## Abstract

**Background/objectives:**

*Staphylococcus aureus* (*S. aureus*) can cause life-threatening infections, with colonization often preceding infection. Understanding the determinants of *S. aureus* carriage may improve infection prevention. While smoking has been associated with *S. aureus* carriage, studies on smokeless tobacco products remain scarce. This study aimed to investigate whether the use of snuff (Swedish snus), a smokeless tobacco product, is associated with *S. aureus* carriage in adolescents.

**Design:**

We used data from Fit Futures 1, a population-based cross-sectional study including 1,038 participants (93% attendance).

**Methods:**

A total of 457 boys and 443 girls had data on snuff use and two nasal and throat swab cultures for *S. aureus* carriage assessment. Snuff use was defined as occasional or daily use. Logistic regression analysis was used to examine the association between snuff use and *S. aureus* carriage, with odds ratios (ORs) adjusted for known risk factors.

**Results:**

Snuff use was associated with a 64% higher odds of nasal carriage (95% CI = 1.18-2.26; carriage defined as one or two positive nasal cultures) compared to non-use. In sex-stratified analyses, this association was observed only among girls, with an adjusted OR of 1.62 (95% CI = 1.03-2.55; carriage defined as two positive nasal cultures) and an adjusted OR of 1.99 (95% CI = 1.25-3.16; carriage defined as one or two positive nasal cultures). Among girls using snuff, the adjusted OR for *S. aureus* throat carriage was 1.66 (95% CI 1.06-2.59; carriage defined as two positive throat cultures) compared to non-users.

**Conclusion:**

We identified an association between snuff use and *S. aureus* nasal and throat carriage among adolescent girls. Girls who used snuff had higher odds of *S. aureus* nasal (62%) and throat (66%) carriage compared to female non-users. Futures longitudinal studies are needed to clarify whether the observed associations reflect casual relationships.

## Introduction

*Staphylococcus aureus* (*S. aureus*) is one of the most potent human bacterial pathogens and can cause life-threatening infections.^1^ However, 20-30% of the general adult population carry *S. aureus* in the anterior nares as part of their normal flora, with higher carriage rates observed among men and younger age groups.^2-6^ Studies among various patient populations have shown that nasal carriers have an increased risk of *S. aureus* infections and are frequently autoinfected with their own bacterial strain.^7^ Because colonization precedes infection, and *S. aureus* infections are associated with substantial morbidity and mortality, carriage represents a major public health concern.^8,9^

To reduce the risk of infections, hospitals have implemented measures to eradicate or at least suppress nasal carriage prior to surgery and invasive procedures.^10^ *S. aureus* also colonizes other anatomical sites including the throat, though the role of this reservoir in transmission and infection remains less well understood.^11^ Nasal decolonization is typically followed by subsequent decolonization of the perineum, pharynx, and axillae, reinforcing the assumption that the nose serves as the primary site of *S. aureus* colonization.^11^

Prevention of *S. aureus* infections and death is a major clinical and public health challenge. Identifying modifiable determinants of *S. aureus* carriage is therefore essential, as effective prevention strategies rely on understanding factors that may be amenable to intervention. Colonization likely results from an interplay between host and microbe. For instance, multiple adaptive bacterial mutations associated with *S. aureus* colonization have been demonstrated.^12^Cross-sectional studies have found that high serum glucose levels and low serum vitamin D levels are associated with a higher prevalence of *S. aureus* nasal carriage.^4,5^ Furthermore, young women using combination hormonal contraceptives have been found to have higher nasal carriage rates than non-users, and carriage has also been linked to circulating testosterone in women.^13,14^

Cigarette smoking has been negatively associated with *S. aureus* nasal carriage in some observational studies,^5^ while other studies have reported a positive association between smoking and nasal carriage,^15^ or as having no association.^16^ It has been hypothesized that a link with cigarette smoking may be due to effects on both microbe and host. Lacoma et al showed that cigarette smoke exposure induces strain-specific growth inhibition of *S. aureus*, while also enhancing biofilm formation, as well as increased invasion and persistence in bronchial alveolar epithelial cells.^17^ In an in-vitro study investigating the nicotine component of cigarette smoke, Shi et al found that nicotine treatment enhanced *S. aureus* adherence and biofilm formation but inhibited bacterial virulence.^18^ Cigarette smoking affects both innate and adaptive immune responses, thereby increasing the risk of infections.^19^ Cole et al reported that healthy smokers had higher nasal *S. aureus* loads than healthy non-smokers and that smoking cessation improved innate host immune responses and increased the clearance rate of *S. aureus*.^20^ However, it remains unclear whether direct exposure to cigarette smoke or indirect exposure to tobacco-derived chemicals through systemic uptake plays a more significant role in *S. aureus* nasal carriage. Notably, studies examining the impact of smokeless tobacco products on *S. aureus* carriage are largely lacking.

In Norway, the use of the smokeless tobacco product “Swedish snus,” a moist snuff placed under the lip, has a long tradition and is legally available to individuals aged 18 years and older.^21^ According to Statistics Norway, use of snuff has increased over the past decade, with the proportion of daily users aged 16-24 years rising from 18% in 2011 to 21% in 2024.^22^ Its speculated that this increase in use is due to availability and the perception of low risk with use.^23^ Compared to smoking, research on the potential health effects of smokeless tobacco remains limited. To the best of our knowledge, no studies have investigated a possible association between snuff use and *S. aureus* carriage. Given that snuff components are absorbed through the oral mucosa and enter systemic circulation, it is plausible that snuff use may be a determinant for *S. aureus* carriage both in the nasal cavity and oropharynx. The aim of this study was to investigate the potential association between snuff use and nasal and throat carriage of *S. aureus* in a general adolescent population in Northern Norway.

## Material and Methods

### Population and Study Design

The study population consisted of participants from the Fit Futures 1 study (FF1).^24^ Conducted in 2010-2011, FF1 invited all first-year upper-secondary school students from the municipalities of Tromsø and Balsfjord in Northern Norway to participate in a health and lifestyle examination. A total of 1,038 students attended (508 girls and 530 boys), corresponding to a 93% attendance rate. FF1 participants attended a half-day visit at the Clinical Research Unit, University Hospital of North Norway. During this visit, they provided information on alcohol use, snuff use, smoking, atopic eczema, physical activity and hormonal contraceptives through a self-administered an self-reported electronic questionnaire. Additionally, trained nurses conducted interviews about medications, chronic disease, social network and menstruation/pregnancy. As well as preforming microbiological sampling, blood sampling, and general clinical examinations, including height and weight measurements for body mass index (BMI) calculation (kg/m^2^).^25^

**Figure 1.**
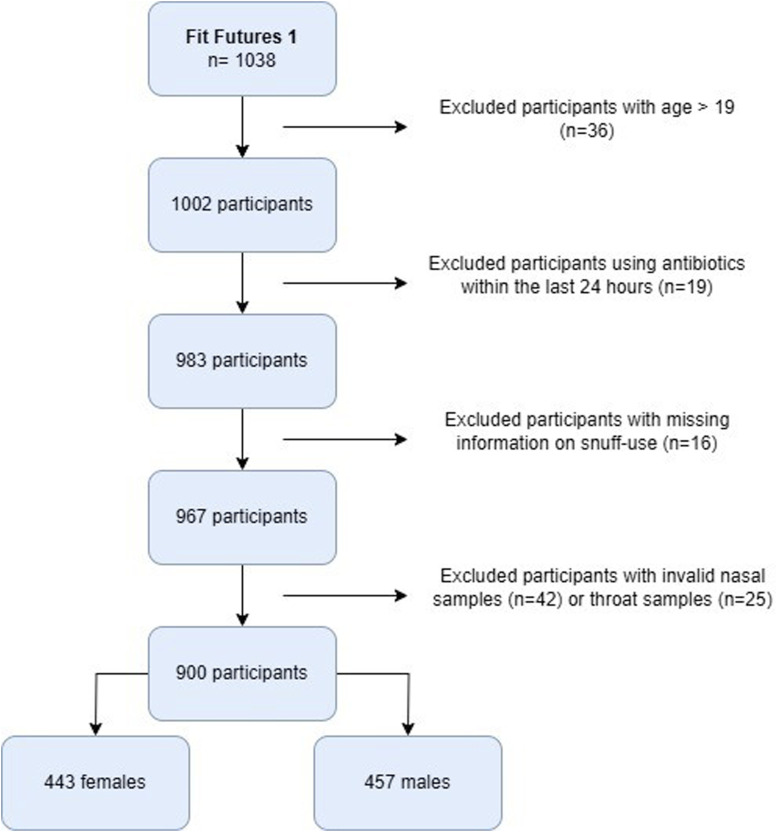
The study population. Fit Futures 1

We excluded 36 participants due to an age > 19 years, in accordance with the World Health Organization’s definition of adolescents as individuals aged 10-19 years.^26^ Additionally, 19 participants were excluded due to antibiotic intake within the 24 hours preceding nasal and throat swabbing. Furthermore, 83 participants were excluded due to missing data on snuff use and/or nasal and throat swab samples (Figure 1). Excluded participants did not differ in terms of age, BMI, glycated hemoglobin (HbA1c), atopic eczema, smoking, alcohol use or physical activity compared to study participants.

### Detection of *S. aureus* Colonization and Carriage

To detect *S. aureus* carriage, samples were collected during the half-day hospital visit and repeated approximately one week later at the school, both times by trained health personnel. Swabs were taken from the anterior nares and the surface of both tonsils with NaCl-moistened sterile swabs. The swabs were enriched in Bacto Staphylococcus medium broth (Difco laboratories, Sparks, MD, USA) and incubated for 18-24 hour at 37 °C. One drop of enrichment broth was streaked on blood agar, chromID *S. aureus* and chromID™ MRSA agars (bioMérieux, Marcy l’Etoile, France) and incubated for 48 hours at 35°C. Colony morphology on the agar plates served as the basis for further *S. aureus* identification. Suspected positive colonies were confirmed as *S. aureus* using the Staphaurex Plus agglutination test (Murex Diagnostic Ltd, Dartford, UK). No methicillin-resistant*Staphylococcus aureus* (MRSA) isolates were detected. Based on the culturing results, *S. aureus* carriage was classified into three categories: non-carriers (two negative swabs), intermittent carriers (one positive swab), and persistent carriers (two positive swabs). In the logistic regression models, we used a dichotomous *S. aureus* outcome variable; “Persistent carriers” versus “Others” (including non- and intermittent carriers), representing a modified application of the *S. aureus* carriage classification proposed by van Belkum et al.^27^ Additionally, we applied an alternative dichotomization, categorizing participants as “Carriers” (at least one positive swab) and “Non-carriers” (two consecutive negative swabs).

### Smokeless Tobacco Use Assessment

Smokeless tobacco use was assessed through an electronic questionnaire, which included the question: “Do you use snuff?” with response options “No, never”, “Yes, sometimes”, “Yes, daily”. For the statistical analysis, snuff use was recoded into a dichotomous variable with the categories “No snuff use/non-user” and “Snuff use/user”.

### Statistical Analysis

**Figure 2.**
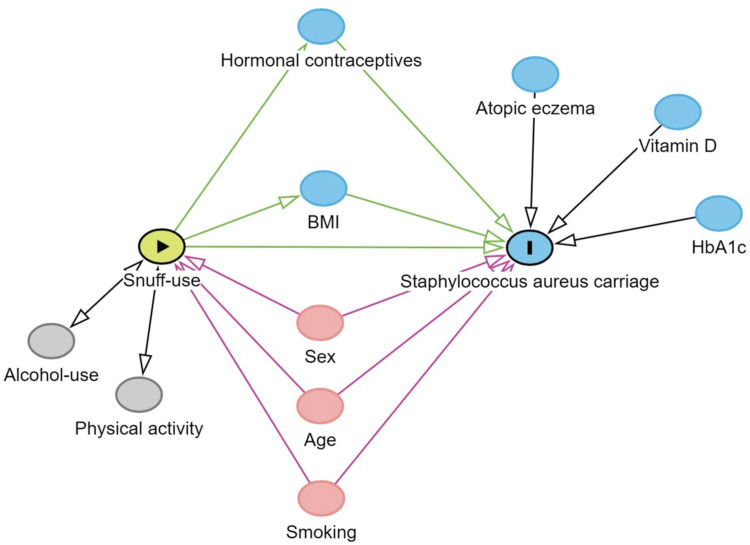
Directed acyclic graph for the association between snuff-use and Staphylococcus aureus carriage

To investigate the association between snuff use and nasal and throat carriage of *S. aureus*, we used descriptive analyses and logistic regression models. Differences in *S. aureus* carriage prevalence between snuff users and non-users were assessed using chi-square test. Logistic regression analysis was conducted to estimate the odds ratio (OR) for *S. aureus* nasal and throat carriage among snuff users compared to non-users adjusting for potential confounding factors. Model selection was performed using DAGitty software (Figure 2).^28^ Because the prevalence of *S. aureus* is well known to differ between women and men, and because the pattern of *S. aureus* carriage in relation to snuff use also varied by sex in our dataset, we chose to stratify the analyses by sex, in addition to presenting results for the total population, despite the test for interaction not reaching statistical significance. Missing data was excluded from the analysis. Hosmers-Lemeshow goodness of fit is reported for all statistical models. All statistical analyses were done using Stata/MP 18.5 for Macintosh, and statistical significance was set at p < 0.05.

## Results

The FF1 study population had a mean age of 16.2 years, and snuff use was more prevalent among boys than girls. Daily snuff use was reported by 27.6% of boys and 17.8% of girls. In contrast, a higher proportion of girls reported sporadic snuff use compared to boys (14.7 % vs. 12.0%, respectively) (Table 1).

**Table 1.** Characteristics of the Study Population in the Fit Futures 1 Study. Figures are Means (Standard Deviations) and Numbers (Percent). Numbers May Vary due to Missing Values

​	Total, N=900	Girls, N=443	Boys, N=457
**Age at screening, years**	16.2 (0.6)	16.2 (0.6)	16.2 (0.6)
**BMI, kg/m** ^**2**^	22.5 (4.2)	22.5 (4.1)	22.5 (4.3)
**HbA1c in EDTA whole blood, %**	5.3 (0.3)	5.3 (0.3)	5.3 (0.3)
**25-hydroxyvitamin D in serum, nmol/L**	47.2 (22.9)	54.4 (23.3)	40.6 (20.6)
**Atopic eczema**			
Yes	134 (15.0%)	79 (18.0%)	55 (12.1%)
No	759 (85.0%)	360 (82.0%)	399 (87.9%)
**Use of hormonal contraceptives**			
Combined contraceptive	​	130 (29.3%)	​
Progesterone only contraceptive		18 (4.1%)	
No hormonal contraceptive		295 (66.6%)	
**Smoking**			
No, never	704 (78.3%)	352 (79.6%)	52 (77.0%)
Sometimes	161 (17.9%)	73 (16.5%)	88 (19.3%)
Daily	34 (3.8%)	17 (3.9%)	17 (3.7%)
**Snuff use**			
No, never	575 (63.9%)	299 (67.5%)	276 (60.4%)
Sometimes	120 (13.3%)	65 (14.7%)	55 (12.0%)
Daily	205 (22.8%)	79 (17.8%)	126 (27.6%)
**Alcohol use**			
Never	254 (28.3%)	106 (23.9%)	148 (32.5%)
Once per month or less	369 (41.1%)	201 (45.4%)	168 (36.9%)
2-4 times per month or more	275 (30.6%)	136 (30.7%)	139 (30.6%)
**Physical activity** ^*^			
Inactive/sedentary	201 (22.3%)	66 (14.9%)	135 (29.5%)
Low	289 (32.1%)	175 (39.5%)	114 (25.0%)
Medium	230 (25.6%)	130 (29.4%)	100 (21.9%)
Hard	180 (20.0%)	72 (16.2%)	108 (23.6%)

BMI = body mass index; HbA1c = glycated haemoglobin.

*Inactive/sedentary = Reading, watching TV or other sedentary activity; Low = Walking, cycling or other forms of exercise at least 4 hours a week; Medium = Participation in recreational sports or heavy outdoor activity; Hard = Participation in hard training or sports regularly.

In the total study population, the prevalence of nasal *S. aureus* carriage was 58.8% based on the “alternative” carrier definition (at least one positive sample, 529/900, Table 2) with persistent nasal carriage observed in 45.1% of the participants based on the “modified van Belkum” classification (two positive samples, 406/900). The prevalence of persistent *S. aureus* nasal carriage was 43.0% among non-users of snuff and 48.9% among users (p = 0.084). In sex-stratified analysis, a similar pattern was observed only among girls. When applying the alternative carrier definition, the prevalence of nasal carriage was 55.1% among non-users of snuff, compared to 65.2%, among users (p = 0.01). Among female non-users, the prevalence of nasal carriage was 49.1%, whereas it was 62.5% among female snuff users (p = 0.01).

**Table 2.** Repeated Nasal Swab Samples. *Staphylococcus aureus* Culturing Results and Carrier Status by Snuff Use (N and Percent in Brackets). The Fit Futures 1 Study

​	Total population (N = 900)				Girls (N = 443)				Boys (N = 457)			
Culturing results:	Two neg.	One pos. One neg.	Two pos.	P*	Two neg.	One pos. One neg.	Two pos.	P*	Two neg.	One pos. One neg.	Two pos.	P*
No snuff use	258 (44.9)	70 (12.2)	247 (42.9)	.01	152 (50.8)	40 (13.4)	107 (35.8)	.03	106 (38.4)	30 (10.9)	140 (50.7)	.33
Snuff use	113 (34.8)	53 (16.3)	159 (48.9)	​	54 (37.5)	27 (18.7)	63 (43.8)	​	59 (32.6)	26 (14.4)	96 (53.0)	​
Total	371 (41.2)	123 (13.7)	406 (45.1)	​	206 (46.5)	67 (15.1)	170 (38.4)	​	165 (36.1)	56 (12.3)	236 (51.6)	​

^Modified van Belkum definition^	^Others^	^Persistent carriers^	^P*^	^Others^	^Persistent carriers^	^P*^	^Others^	^Persistent carriers^	^P*^			
^No snuff use^	^328 (57.0)^	^247 (43.0)^	^.08^	^192 (64.2)^	^107 (35.8)^	^.11^	^136 (49.3)^	^140 (50.7)^	^.62^			
^Snuff use^	^166 (51.1)^	^159 (48.9)^	​	^81 (56.3)^	^63 (43.7)^	​	^85 (47.0)^	^96 (53.0)^				​
^Total^	^494 (54.9)^	^406 (45.1)^	​	^273 (61.6)^	^170 (38.4)^	​	^221 (48.4)^	^236 (51.6)^				​

^Alternative carrier definition^	^Non-carriers^	^Carrier^	^P*^	^Non-carrier^	^Carrier^	^P*^	^Non-carrier^	^Carrier^	^P*^			
^No snuff use^	^258 (44.9)^	^317 (55.1)^	^.01^	^152 (50.8)^	^147 (49.1)^	^.01^	^106 (38.4)^	^170 (61.6)^	^.21^			
^Snuff use^	^113 (34.8)^	^212 (65.2)^	​	^54 (37.5)^	^90 (62.5)^	​	^59 (32.6)^	^122 (67.4)^				​
^Total^	^371 (41.2)^	^529 (58.8)^	​	^206 (46.5)^	^237 (53.5)^	​	^165 (36.1)^	^292 (63.9)^				​

*P-value, Chi-square test for difference in carrier status between non-users and users of snuff.

The prevalence of throat carriage in the total study population was 79.1% (712/900) with persistent carriage observed in 50.8% (457/900, Table 3). Among girls who never used snuff, the prevalence of persistent *S. aureus* throat carriage was 38.5%, compared to 50.0% among girls who used snuff (p = 0.02, Table 3). No statistically significant difference was observed in the prevalence of persistent throat carriage between boys who never used snuff (60.9%) and those who used snuff (56.4%).

**Table 3.** Repeated Throat Swab Samples. *Staphylococcus aureus* Culturing Results and Carrier Status by Snuff Use (N and Percent in Brackets). The Fit Futures 1 Study

​	Total population (N = 900)				Girls (N = 443)				Boys (N = 457)			
Culturing results:	Two neg.	One pos. One neg.	Two pos.	P*	Two neg.	One pos. One neg.	Two pos.	P*	Two neg.	One pos. One neg.	Two pos.	P*
No snuff use	126 (21.9)	166 (28.9)	283 (49.2)	.42	83 (27.7)	101 (33.8)	115 (38.5)	.05	43 (15.6)	65 (23.6)	168 (60.9)	.57
Snuff use	62 (19.1)	89 (27.4)	174 (53.5)	​	28 (19.4)	44 (30.6)	72 (50.0)	​	34 (18.8)	45 (24.9)	102 (56.3)	​
Total	188 (20.9)	255 (28.3)	457 (50.8)	​	111 (25.1)	145 (32.7)	187 (42.2)	​	77 (16.9)	110 (24.1)	270 (59.1)	​

^Modified van Belkum definition^	^Others^	^Persistent carriers^	^P*^	^Others^	^Persistent carriers^	^P*^	^Others^	^Persistent carriers^	^P*^			
^No snuff use^	^292 (50.8)^	^283 (49.2)^	^.21^	^184 (61.5)^	^115 (38.5)^	^.02^	^108 (39.1)^	^168 (60.9)^	^.34^			
^Snuff use^	^151 (46.5)^	^174 (53.5)^	​	^72 (50.0)^	^72 (50.0)^	​	^79 (43.6)^	^102 (56.4)^				​
^Total^	^443 (49.2)^	^457 (50.8)^	​	^256 (57.8)^	^187 (42.2)^	​	^187 (40.9)^	^270 (59.1)^				​

^Alternative carrier definition^	^Non-carriers^	^Carriers^	^P*^	^Non-carriers^	^Carriers^	^P*^	^Non-carriers^	^Carriers^	^P*^			
^No snuff use^	^126 (21.9)^	^449 (78.1)^	^.32^	^83 (27.8)^	^216 (72.2)^	^.06^	^43 (15.6)^	^233 (84.4)^	^.37^			
^Snuff use^	^62 (19.1)^	^263 (80.9)^	​	^28 (19.4)^	^116 (80.56)^	​	^34 (18.8)^	^147 (81.2)^				​
^Total^	^188 (20.98)^	^712 (79.1)^	​	^111 (25.1)^	^332 (74.9)^	​	^77 (16.9)^	^380 (83.2)^				​

*P-value, Chi-square test for difference in carrier status between non-users and users of snuff.

In the total study population, snuff use was associated with a 52% higher odds of *S. aureus* nasal carriage (OR =1.52, 95% CI = 1.15-2.02, results not presented in tables) compared to non-use. After further adjustment for smoking, age and sex the OR was 1.64 (95% CI = 1.18-2.26, Table 4). A significant association between snuff use and *S. aureus* nasal carriage was observed among girls, with an adjusted OR of 1.99 (95% CI = 1.25-3.16) compared to non-use. The same association was found for persistent carriage among girls with an OR of 1.62 (95% CI = 1.03-2.55). Among boys, no association was found between snuff use and *S. aureus* nasal carriage.

**Table 4.** Associations Between Snuff Use and *Staphylococcus aureus* Nasal Carrier State; Odds Ratios (OR) and 95% Confidence Intervals (95% CI) From Multivariable Logistic Regression Analysis. The Fit Futures 1 Study

​		Nasal carriage^†^ (≥1 positive sample)			Persistent nasal carriage^††^ (2 positive sample)		
		Total population (N=900)^a^	Girls (N=443)^b^	Boys (N=457)^c^	Total population (N=900)^d^	Girls (N=443)^e^	Boys (N=457)^f^
		OR (95% CI)	OR (95% CI)	OR (95% CI)	OR (95% CI)	OR (95% CI)	OR (95% CI)
Snuff use	Non-user	1.0 (ref)	1.0 (ref)	1.0 (ref)	1.0 (ref)	1.0 (ref)	1.0 (ref)
	User	**1.64 (1.18-2.26)**	**1.99 (1.25-3.16)**	1.33 (0.83-2.11)	1.33 (0.98-1.83)	**1.62 (1.03-2.55)**	1.11 (0.71-1.72)
Smoke	Non-user	1.0 (ref)	1.0 (ref)	1.0 (ref)	1.0 (ref)	1.0 (ref)	1.0 (ref)
	User	0.80 (0.55-1.16)	0.69 (0.40-1.18)	0.94 (0.55-1.61)	0.82 (0.56-1.17)	0.68 (0.39-1.16)	0.98 (0.59-1.64)
Age	years	1.20 (0.96-1.52)	**1.63 (1.14-2.34)**	0.94 (0.70-1.28)	1.04 (0.84-1.30)	1.21 (0.87-1.68)	0.92 (0.69-1.23)
Sex	Girls	1.0 (ref)	​	​	1.0 (ref)	​	​
	Boys	**1.52 (1.16-1.99)**	​	​	**1.71 (1.31-2.23)**	​	​

^†^Observations with one or two *S. aureus* positive cultures defined as carriers.

^††^Observations with two *S. aureus* positive cultures defined as carriers.

Goodness of fit: ^a^p = 0.302, ^b^p = 0.448, ^c^p = 0.483, ^d^p = 0.871, ^e^p = 0.762, ^f^p = 0.807. Statistically significant values (p < 0.05) are shown in bold.

In the multivariable logistic regression analysis, female participants using snuff had an increased odds of persistent throat carriage of 66% (OR = 1.66, 95% CI= 1.06-2.59, Table 5). No statistically significant association was observed between snuff use and persistent *S. aureus* throat carriage among boys or in the total population.

**Table 5.** Associations Between Snuff Use and *Staphylococcus aureus* Throat Carrier State; Odds Ratios (OR) and 95% Confidence Intervals (95% CI) From Multivariable Logistic Regression Analysis. The Fit Futures 1 Study

​		Throat carriage^†^ (≥1 positive sample)			Persistent throat carriage^††^ (2 positive sample)		
		Total population (N=900)	Girls (N=443)	Boys (N=457)	Total population (N=900)	Girls (N=443)	Boys (N=457)
		OR (95% CI)	OR (95% CI)	OR (95% CI)	OR (95% CI)	OR (95% CI)	OR (95% CI)
Snuff use	Non-user	1.0 (ref)	1.0 (ref)	1.0 (ref)	1.0 (ref)	1.0 (ref)	1.0 (ref)
	User	1.16 (0.79-1.71)	1.56 (0.91-2.66)	0.83 (0.46-1.47)	1.11 (0.81-1.53)	**1.66 (1.06-2.59)**	0.75 (0.48-1.17)
Smoke	Non-user	1.0 (ref)	1.0 (ref)	1.0 (ref)	1.0 (ref)	1.0 (ref)	1.0 (ref)
	User	0.97 (0.62-1.52)	1.06 (0.57-1.97)	0.93 (0.47-1.79)	1.05 (0.73-1.51)	0.92 (0.55-1.56)	1.25 (0.74-2.09)
Age	years	1.10 (0.83-1.45)	1.31 (0.86-1.97)	0.93 (0.64-1.36)	1.08 (0.87-1.35)	1.22 (0.88-1.69)	0.97 (0.72-1.31)
Sex	Girls	1.0 (ref)	​	​	1.0 (ref)	​	​
	Boys	**1.65 (1.18-2.28)**	​	​	**1.98 (1.52-2.58)**	​	​

^†^Observations with one or two *S. aureus* positive cultures defined as carriers.

^††^Observations with two *S. aureus* positive cultures defined as carriers (modified Van Belkum).

Goodness of fit: ^a^p = 0.146, ^b^p = 0.254, ^c^p = 0.362, ^d^p = 0.038, ^e^p = 0.027, ^f^p = 0.478.

A sensitivity analysis including snuff-use only, smoking only and dual-use (participants reporting smoking and snuff use) showed an increased odds of nasal carriage for the snuff-only group. Participants reporting only smoking had lower odds of nasal carriage compared to non-users, though not statistically significant. Dual-use was not statistically significantly associated with nasal carriage, representing the conflicting effects of smoking and snuff-use (Supplementary table 1). No similar association was shown for the sensitivity analysis for throat carriage (Supplementary table 2).

## Discussion

In this population-based cross-sectional study, we identified an association between snuff use and *S. aureus* carriage among adolescent girls. Our findings indicate that girls who used snuff either occasionally or daily had 99% increased odds of nasal carriage of *S. aureus* compared to non-users. Additionally, they exhibited a 62% higher odds of persistent nasal carriage and a 66% higher odds of *S. aureus* throat carriage.

In our study, the association between snuff use and *S. aureus* carriage was observed only among girls, while no similar association was found in boys. The reason for this discrepancy remains speculative. Male sex is a well-established risk factor for *S. aureus* nasal carriage, as demonstrated in several epidemiological studies, including the Fit futures cohort.^13^ Additionally, sex steroid hormones are known to regulate immune responses,^29^ and circulating sex-steroid levels have been identified as determinants for *S. aureus* carriage.^5,14^ Studies have shown that smoking is associated with lower estrogen levels and higher testosterone levels in females.^30,31^ If smokeless tobacco exerts a similar effect on sex steroid hormones, this could be a plausible explanation for why the association between snuff use and *S. aureus* carriage was observed only in females. However, it remains unclear whether the increased prevalence of *S. aureus* nasal and throat carriage among snuff users is directly caused by tobacco exposure or by other factors associated with snuff use, such as shared behavioral or environmental exposures. The use of snuff involves frequent hand-to-mouth contact and sharing of snuff containers, which may facilitate *S. aureus* transmission. This theory is strengthened by the conflicting results between snuff-use and smoking, also demonstrated in other studies.^5^ A 2016 study found that some smokeless tobacco products contained *S. aureus*,^32^ supporting the hypothesis that snuff use may be a risk factor for *S. aureus* carriage. Nevertheless, further research, including larger population-based prospective studies, is needed to establish a causal relationship.

To the best of our knowledge, no previous studies have investigated the association between smokeless tobacco use and *S. aureus* nasal or throat carriage. One study demonstrated that smokeless tobacco extract (STE) at low concentrations enhanced the production of cytokines.^33^ However, findings from Hasseus et al suggest that water-soluble extracts from Swedish moist snuff significantly inhibited T-cell proliferation induced by accessory cells from rats,^34^ indicating an immunosuppressive effect. Shi et al reported that nicotine treatment enhances *S. aureus* biofilm formation by promoting initial attachment and extracellular DNA release, while simultaneously inhibiting *S. aureus* virulence.^18^ The existing research presents conflicting evidence, making it difficult to draw definitive conclusions regarding the effects of smokeless tobacco on the immune response. Given our findings of an association between smokeless tobacco use and a higher prevalence of *S. aureus* colonization, it is plausible that smokeless tobacco may inhibit the innate immune response or promote bacterial adherence and growth.

This study has several important strengths, but also limitations that should be considered when interpreting the findings. Strengths of this study include the high response rate (93% attendance), which likely reduces selection bias. The prevalence of the main predictor snuff-use is similar compared to the national prevalence.^22^ We therefore believe the findings to be generalizable to adolescents with similar sosioeconomic backgrounds in comparable societies where smokeless tobacco is available.

The use of two repeated nasal and throat cultures collected approximately one week apart represents a methodological strength. This repeated sampling design improves the reliability of carriage classification compared with single-sample studies. Participants with two culture-positive samples from a single site can be classified with greater confidence as persistent or *S. aureus*–dominant carriers^27,35^ Our finding of an association between snuff use and any *S. aureus* positive culture from one site may indicate that snuff users have a higher likelihood of both persistent and transient colonization. Alternatively, limited sampling, particularly at school, may have led to some misclassification of persistent carriers.

The high prevalence of *S. aureus* carriage observed in our study is partly attributable to the use of enrichment methods and should be considered when comparing our results with other studies.^13^ Access to a large dataset with a wide range of variables allowed adjustment for several known risk factors for *S. aureus* nasal and throat carriage. As a population-based study, the findings are largely representative of the general adolescent population.

Nevertheless, some limitations must be acknowledged. The study was conducted within a narrow age range, and the results cannot be directly generalized to other age groups without further research. Another limitation is that snuff use was self-reported. Since the sale of snuff and tobacco products to individuals under 18 years of age is illegal in Norway, underreporting of snuff use is possible. However, any misclassification of snuff use is likely to be non-differential, meaning that it would affect both carriers and non-carriers of *S. aureus* equally, potentially attenuating the observed associations. Furthermore, we did not collect data on the average number of snuff portions consumed per week among users, which precluded assessment of a potential dose-response relationship. In addition, the questionnaire was not a previously validated instrument, and no formal pilot study with predefined testing procedures was conducted. However, previous research among adolescents in similar age groups has demonstrated high sensitivity and specificity of self-reported snus use, suggesting that such measures are generally reliable.^36^ Nonetheless, the lack of formal validation should be considered when interpreting the findings.

The logistic regression analysis was adjusted for known risk factors for *S. aureus* carriage, including sex, age and smoking. However, residual confounding due to unmeasured variables cannot be ruled out, including social network.^37^ A formal power calculation was not performed because the analyses were based on the entire Fit Futures cohort rather than on a sample drawn from a larger source population. The sample size was therefore fixed and not determined by an a priori design consideration. In this setting, statistical precision is more appropriately evaluated through the reported effect estimates and their 95% confidence intervals. Post hoc power calculations would not provide additional meaningful information beyond these measures.

## Conclusion

Our findings indicate a higher risk of *S. aureus* colonization among girls who use snuff. This supports the hypothesis that smokeless tobacco use may have negative health effects that are not yet fully understood. These findings are relevant for public health education, highlighting the potential risks associated with smokeless tobacco use. Additionally, our study contributes to the understanding of possible determinants of *S. aureus* carriage. This knowledge is valuable, as it may offer new perspectives on controlling the *S. aureus* reservoir and preventing *S. aureus*-related diseases in the population. However, future studies are needed to determine whether a causal relationship exists between smokeless tobacco use and *S. aureus* carriage.

## Supplemental Material

Supplemental Material -Sex-Specific Associations Between Smokeless Tobacco Use and *Staphylococcus aureus* Carriage in Norwegian Adolescents Supplemental Material for Sex-Specific Associations Between Smokeless Tobacco Use and *Staphylococcus aureus* Carriage in Norwegian Adolescents by Dina B. Stensen, Anna Karlsen, Gunnar Skov Simonsen, and Anne-Sofie Furberg in Tobacco Use Insights.

## Data Availability

The data that support the findings of this study are available from the Fit Futures study, but restrictions apply to the availability of these data, which were used under license for the current study, and so are not publicly available. Data are however available upon request from the Fit Futures study, fitfutures@uit.no.
